# Metal-siloxanes derived bio-inspired superhydrophobicity and nitric oxide generation for anti-biofouling clinical applications

**DOI:** 10.1039/d6tb01036a

**Published:** 2026-07-23

**Authors:** Annalise D. M. Tucker, Ekrem Ozkan, Sarah N. Wilson, Arpita Shome, Hitesh Handa, Elizabeth J. Brisbois

**Affiliations:** a School of Chemical, Materials and Biomedical Engineering, College of Engineering, University of Georgia 302 East Campus Road Athens Georgia 30602 USA ejbrisbois@uga.edu +1 706-542-1243; b Department of Pharmaceutical and Biomedical Sciences, College of Pharmacy, University of Georgia Athens Georgia 30602 USA

## Abstract

Bio-inspired superhydrophobic surfaces are widely investigated for antibiofouling applications; however, they lack intrinsic bioactivity and provide passive resistance without eliminating planktonic or surface-adhered microbes. In this work, superhydrophobicity is integrated with bioactive nitric oxide (NO) generation from physiological levels of *S*-nitrosothiols (RSNOs) to provide combined antibiofouling under biologically relevant conditions. An optimized combination of non-fluorinated organosilane-modified micro- and nanosized zinc oxide (ZnO) and copper (Cu) particles within a siloxane matrix yields a durable coating on a polydimethylsiloxane substrate. The coating exhibits a surface roughness of 353 ± 26 nm and maintains water repellence following sand abrasion, water-jet, tape-peeling, and scratch test, with a contact angle >150° and hysteresis <2°. Upon exposure to physiological levels of *S*-nitrosoglutathione (GSNO) and glutathione (GSH), the coating demonstrates an 86.4 ± 4.0% increase in NO generation relative to controls, with fluxes matching endothelial NO production. The cytocompatible coating shows negligible metal leaching and excellent antibacterial activity, achieving ∼ 99.99% and 99.79% reductions in surface-adhered *S. aureus* and *E. coli*, respectively, and 89.43% and 69.80% reductions in planktonic *S. aureus* and *E. coli*, respectively. This work establishes a scalable strategy integrating non-adhesive superhydrophobicity with catalytic NO generation and demonstrates effective antimicrobial performance under physiologically relevant NO-generating conditions.

## Abstract 2

A durable superhydrophobic ZnO/10Cu coating integrates passive bacterial repulsion with physiological nitric oxide generation, reducing surface bacterial attachment while actively eliminating planktonic bacteria.

## Graphical abstract

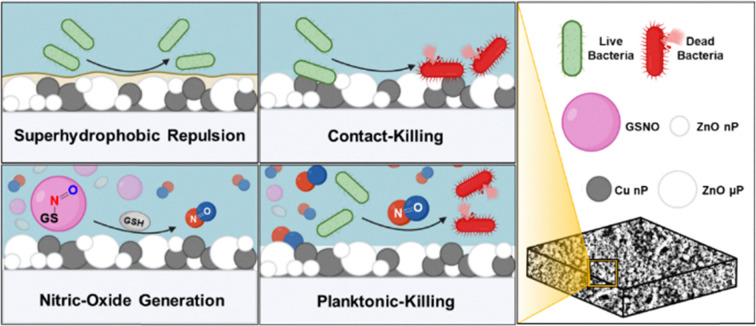

## 1. Introduction

Medical device-associated infections can be initiated by the adhesion of a single bacterium and contribute to approximately 1.7 million hospital-acquired infections and 99 000 deaths annually.^1^ In the United States, approximately 1 in 31 hospitalized patients contract at least one healthcare-associated infection each day, underscoring the need for improved prevention strategies.^1^ Bacterial adherence to device surfaces can lead to persistent contamination and infection, particularly in the presence of multidrug-resistant strains that limit the effectiveness of systemic antibiotic therapy.^2–5^ These challenges underscore the need for novel, non-antibiotic strategies to prevent bacterial adhesion and subsequent complications.

Effective antibacterial surfaces should exhibit both antibiofouling and bactericidal characteristics. Antibiofouling properties inhibit initial bacterial attachment, whereas bactericidal functionality inactivates or suppresses bacterial growth through chemical or physical mechanisms following contact.^6^ Inspired by the water-repellent properties of the lotus leaf, biomimetic superhydrophobic surfaces have been developed through control of surface topography and chemistry to impart passive antibiofouling behavior.^7–11^ According to the Cassie–Baxter model, a trapped metastable air layer minimizes water–surface contact, thereby reducing adhesion forces and enabling contaminants to be removed by rolling droplets.^12^

Metals have been widely employed in the fabrication of superhydrophobic antibiofouling surfaces, including incorporation of low-surface-energy-functionalized metal–organic frameworks, blending of metal or metal oxide particles into polydimethylsiloxane (PDMS), surface modification with fluorinated or long alkyl chains, and modification of metals prior to PDMS incorporation.^7,8^ While such metal-derived superhydrophobic surfaces can effectively reduce bacterial adhesion, their activity against planktonic bacteria in the surrounding environment is underexplored. When reductions in planktonic bacterial counts are observed, activity is often attributed to metal ion leaching—an effect that may be cytotoxic—yet cytotoxicity and quantitative leaching assessments are frequently omitted. Comprehensive evaluation of planktonic antibacterial activity, quantitative metal leaching, and cytocompatibility is therefore critical for distinguishing true surface-mediated antimicrobial functionality from passive metal release and to ensure the safety of such coatings for medical device applications.

Nitric oxide (NO) is a bioactive signaling molecule with well-documented antimicrobial activity and is a central component of the innate immune response.^13^ Owing to its radical nature and short half-life, NO acts through multiple mechanisms, including damage to vital cellular components, interference with bacterial signaling pathways, and disruption of biofilm formation and stability, thereby reducing bacterial viability and adhesion.^14–16^ NO-generating surfaces typically incorporate catalytic metal species that decompose *S*-nitrosothiols (RSNOs), such as *S*-nitrosoglutathione (GSNO), in the presence of reducing agents, such as glutathione (GSH), producing NO *in situ*.^17–21^ Unlike NO-releasing materials that rely on a finite NO reservoir, NO-generating materials can provide sustained and localized NO production when physiological substrates are available. Few studies have examined bacterial responses under GSNO and GSH conditions in the presence of a NO-generating surface, despite the physiological relevance of this NO-rich environment. To date, integration of NO-generating functionality within superhydrophobic surface architecture has not been reported.

In this work, a metal-siloxane-derived superhydrophobic coating incorporating catalytic metal (Cu) and metal oxide (ZnO) particles is reported to enable localized NO generation under physiologically relevant RSNO/GSH conditions. Localized NO generation is achieved *via* the catalytic conversion of endogenous RSNOs to NO at the coating interface. This platform represents the first integration of superhydrophobic surface architecture with NO-generating functionality within a single coating. The integration of engineered surface topography, low-surface-energy chemistry, and catalytic NO generation provides a dual-function strategy that both prevents bacterial adhesion and actively inhibits microorganisms. Antifouling performance, NO generation, metal leaching behavior, cytocompatibility, and antibacterial activity against both adherent and planktonic bacteria are evaluated using multiple complementary, physiologically relevant vascular-mimicking models.

## 2. Materials and methods

### 2.1. Materials

Zinc oxide (ZnO) nanoparticles (10–30 nm) and copper (Cu) nanoparticles (40–60 nm) were sourced from Skyspring Nanomaterials Inc. (Houston, USA), while ZnO microparticles (<5 µm) and other materials such as octadecyl(trimethoxy)silane (ODTMS), *n*-hexane, a mixture of isomers, sodium nitrite (NaNO_2_), hydrochloric acid (HCl), dimethyl sulfoxide (DMSO), sodium chloride, potassium chloride, sodium phosphate monobasic, sodium phosphate dibasic, Copper Assay kit (MAK127), and Luria–Bertani (LB) broth and agar were acquired from Sigma Aldrich (St. Louis, MO). Zinc assay kit (ab176725) was obtained from abcam (Cambridge, MA). Sylgard-184 polydimethylsiloxane base and curing agent (PDMS) were obtained from Ellsworth Adhesives (USA). Reduced l-glutathione was purchased from GoldBio, Inc (St. Louis, MO). *Staphylococcus aureus* (*S. aureus*) (ATCC 6538) and 3T3 mouse fibroblast cells (ATCC CRL-1658) were procured from American Tissue Culture Collection (ATCC, Manassas, VA, USA). Dulbecco's modified Eagle's medium (DMEM), penicillin–streptomycin (PS), and Ethylenediaminetetraacetic acid (EDTA) were sourced from Fischer Scientific. Cell culture-grade calcium and magnesium-free phosphate buffer saline (CMF-PBS, 1×) and 0.25% trypsin were sourced from Corning Incorporated (Corning, NY). Fetal bovine serum (FBS) was sourced from VWR International (Radnor, PA). 3-(4,5-Dimethylthiazol-2-yl)-2,5-diphenyltetrazolium bromide (MTT) reagent sourced from Roche (Basel, CH). DI water was utilized in all appropriate experiments; all chemicals were analytical-grade reagents and utilized without further purification. Phosphate-buffered saline (PBS) was prepared with 139 mM sodium chloride, 2.68 mM potassium chloride, 1.8 mM sodium phosphate monobasic, and 8.2 mM sodium phosphate dibasic. The pH of PBS was verified to be 7.4 at 25 °C using a calibrated pH meter. Calibration was performed with standard buffer solutions of known pH prior to measurement.

### 2.2. Fabrication of superhydrophobic particles and coating solutions

A coating precursor solution was created using specified amounts of ZnO, Cu, ODTMS, *n*-hexane, and DI water, as tabulated in Table 1. In short, ZnO microparticles, ZnO and Cu nanoparticles, ODTMS, and DI water were added to 20 g of *n*-hexane and allowed to mix at 300 rpm for 8 h.

**Table 1:** Ratios of components in coating precursor solution

Formulation^a^	*n*-Hexane (g)	ODTMS (g)	ZnO µp (g)	ZnO np (g)	Cu np (g)	DI (µL)	ZnO : Cu (Ratio)
ZnO	20	0.2	6	6	—	10	100 : 0
ZnO/1Cu	20	0.2	6	6	0.12	10	99 : 1
ZnO/5Cu	20	0.2	6	6	0.60	10	95 : 5
ZnO/10Cu	20	0.2	6	6	1.2	10	90 : 10

a Prior to addition of PDMS in final fabrication step of coating solutions.

To create the final coating solution, 10 wt% Sylgard-184 silicone base and curing agent (10 : 1 ratio of base to curing agent) were added to approximately 20 g of precursor solution. This final coating solution was mixed at 200 rpm for 30 min, vortexed for 1 min, ultrasonicated for 5 min, and vortexed again for 1 min.

Neat Sylgard-184 PDMS was cured and used as the base polymer for film coating. The base films (8 mm in diameter, ∼0.3 mm thick) were dipped into the final coating solution, and any excess solution was gently shaken off. Films were then cured at 100 °C for 2 h to obtain superhydrophobic samples.

Before Sylgard-PDMS addition, the residual precursor solution was allowed to evaporate in a fume-hood overnight, dried at 100 °C for 1 h, and collected to be used in characterization studies as described. Sylgard-184 PDMS base films were weighed before and after dip-coating and curing to determine the mass of the cured coating on the surface, which averaged 6.46 ± 0.98 mg per sample.

Before optimizing final samples with varying weights of Cu, an investigation was conducted on the amount of base metal-oxide components. Superhydrophobic ZnO samples were created with varying amounts of nanoparticles to microparticles to identify concentrations that maintained superhydrophobicity, but minimal surface coating cracking. Representative images are depicted. See Table S1 for the ratios explored.

Unless otherwise noted, experiments involving all coated samples or NO-donor-containing solutions were conducted under light-protected conditions. Samples were routinely shielded from light during preparation, storage, and handling.

### 2.3. Surface characterization

#### 2.3.1. Confirmation of silanization *via* Fourier transform infrared spectroscopy (FTIR)

Fourier transform infrared spectroscopy (FTIR) using a Spectrum 3 spectrometer (PerkinElmer) was used to confirm silanization of nanoparticle and microparticle systems. A quantitative potassium bromide (KBr) method was used with the bare particles and the dried residual precursor solution while the Universal Attenuated Total Reflectance (UATR) FTIR method was used to characterize neat silane. Samples were analyzed in a range of 4000 to 650 cm^−1^ with a resolution of 4 cm^−1^ and a total of 128 scans recorded per sample type. A background spectrum was used before each sample for all FTIR methods.

#### 2.3.2. Antiwetting analysis: water contact angle (WCA) and contact angle hysteresis (CAH)

Antiwetting properties of control PDMS and superhydrophobic sample types were assessed using a contact angle goniometer (Ossila) and image processing software (SURFTENS). To measure the static water contact angle of the surface, 8 mm samples were placed on a stage positioned in front of a camera before a 5 µL droplet of DI water was dispensed onto the surface of the sample. An image of the sample with the water droplet was taken, and the water contact angle was processed using the aforementioned software.

Contact angle hysteresis (CAH) was calculated using the advancing and receding angles the water droplets make with the surface of the samples using eqn (1). Using a Hamilton syringe, a 5 µL droplet was dispensed and withdrawn from the surface of the samples. Videos were recorded and subsequently processed, allowing for analysis of the relative angles. Final data for static water contact angle and hysteresis measurements are reported as the mean ± standard deviation (SD) with *n* ≥ 4 for each sample type.

1 Contact angle hysteresis (°) = Advancing contact angle (°) − Receding contact angle (°)

#### 2.3.3. Surface morphology analysis *via* microscopy

Surface characterization was analyzed *via* scanning electron microscopy (SEM) using a FEI Teneo SEM (FEI Co.) to observe the surface texturization and homogeneity after the metallic dipcoats. Elemental mapping was also conducted on each sample to detect the ZnO and Cu particle dispersion on the surface of the materials using Energy Dispersive X-Ray spectroscopy (EDS, Oxford Instruments). EDS images were analyzed using an Aztec application set to detect Cu, Zn, Carbon, Oxygen, and Silicon on the surface. All images were taken with a magnification of 1000× or 7500× under a 10.00 kV voltage. Before any imaging, the samples were sputter-coated with a 10 nm coating of gold-palladium to enhance readability on the SEM using a Leica sputter coating instrument. Cross-section images of the final sample, ZnO/10Cu, were also taken and analyzed for thickness of the final coating (Fig. S1).

For atomic force microscopy (AFM) analysis, control PDMS and the ZnO/10Cu surface were imaged for their topography and surface roughness measurements. AFM was performed using a Multimode8 Tapping microscope (Bruker) at room-temperature. Topography images were obtained by scanning samples in tapping mode with a scan size of 20 µm by 20 µm, respectively. Scan rates of 0.1 Hz and resolutions of 256 samples per line were used to generate representative images.

LTESPA-V2 cantilever tips (Bruker) with spring constant 42 N m^−1^ were used for all imaging. All images were flattened and plane-fitted using NanoScope Analysis software (Bruker); all films were 2nd order flattened. Roughness measurements were calculated using *n* = 4 of (3 µm × 3 µm) images to represent four different locations per sample type.

#### 2.3.4. Mechanical durability testing

To evaluate superhydrophobic stability, both qualitative and quantitative tests were conducted. Qualitative analyses involved sand abrasion, water jet, tape, and scratch tests as described herein.

A sand-drop test was conducted on the material as per Video S1. In short, the surface was immobilized onto an inclined block, a funnel was placed directly above the surface, and sand (10 g) was dropped from a height of 20 cm. Any residual sand was brushed off the final surface, and the water contact angle after every cycle of abrasion was measured.

The superhydrophobic sample was submerged in DI water (Video S2) to reveal the air plastron layer and placed under a water jet (Video S3) for qualitative observations of surface superhydrophobicity.

The tape-peel test was conducted by placing a piece of tape on top of the surface, adding a 500 g weight for ∼15 s, removing the weight, and peeling the tape from the surface. After the tape was peeled off, a static gun was administered to the surface prior to water contact angle and hysteresis measurements. A razor blade was used to scratch the surface of the material repeatedly. The first cycle was a series of scratches depicted in Video S4; later, the number of cycles equals the number of scratches the surface undergoes. Water contact angle and hysteresis were measured every 10 cycles (every 10 scratches or tape-peels). For detailed *p*-values and statistical significance, please refer to Tables S6 and S7.

### 2.4. Nitric oxide generation

#### 2.4.1. *S*-Nitrosoglutathione synthesis

*S*-Nitrosoglutathione (GSNO) was synthesized from glutathione (GSH) with some modifications to a previously established protocol.^22^ Briefly, reduced glutathione was dissolved in dilute HCl prior to being chilled in an ice bath. Excess sodium nitrite was added to the solution and allowed to cool in the ice bath under stirring for 40 min. After, chilled acetone was added to precipitate the GSNO from the reaction. The precipitate was collected *via* vacuum filtration and washed with cold DI water and additional acetone. The collected filtrate was placed in a desiccator for 24 h before being ground to a fine powder with a mortar and pestle. Powder with a measured purity >95% was used and stored at −20 °C, protected from light until use.

#### 2.4.2. Nitric oxide generation analysis

Nitric oxide release from near physiological levels of GSNO and GSH, 1 and 30 µM, respectively, in the presence of various sample types was quantified.^23,24^ Catalysis and NO generation from fabricated samples were observed in comparison to control PDMS films exposed to the same level of GSNO and GSH. To normalize and ensure the mass of metallic particles remains similar in each formulation, the weight of the films was taken prior to dip-coating and post-curing of the surface coating (Table S9 and eqn (S1)).

Assessment of NO generation involved analysis of accumulated NO and the average NO flux using a Sievers Nitric Oxide Analyzer (280i NOA, Zysense Boulder, CO). Samples were maintained at a physiological temperature of 37 °C, and measurements were performed using an amber reaction chamber and in a dark laboratory environment to minimize light exposure. Initially, amber NOA chambers equipped with accessible ports were filled with 5994 µL of PBS and allowed to stabilize to a baseline. Following stabilization, samples were dropped into the chambers and allowed to stabilize once again. Meanwhile, a solution containing 1 mM GSNO and 30 mM GSH in PBS was prepared. A 6 µL injection of this GSNO/GSH solution was added to the low port access of the chamber using a Hamilton syringe. The resulting NO release was effectively purged from the reaction chamber using a continuous nitrogen sweep gas maintained at a constant flow rate of 200 mL min^−1^, which was subsequently directed into a chemiluminescence reaction chamber for NO quantification. In short, NO reacts with ozone (O_3_) to produce excited nitrogen dioxide (NO_2_*) and oxygen. When the excited nitrogen dioxide eventually returns to its ground state, it emits a photon that can be transformed into a voltage signal and eventual value in parts per billion (ppb). The reaction is depicted in eqn (2) and (3).

2 NO + O_3_ → NO_2_* + O_2_

3 NO_2_* → NO_2_ + *hν*

To evaluate the stability of catalytic NO-generation following prolonged aqueous exposure, ZnO/10Cu samples were incubated in PBS at 37 °C for 72 h. Following incubation, samples were removed from PBS and immediately subjected to the NO-generation protocol described above. Fresh, non-incubated ZnO/10Cu samples were evaluated in parallel as controls. WCA measurements were also performed following PBS incubation to assess retention of the coating's wetting characteristics.

The resulting data in ppb undergoes normalization based upon the sample's surface area and the NOA's calibration constant (mol ppb^−1^ min^−1^). Accumulated NO measurements were determined through area-under-the-curve analysis to quantify the moles of NO released over a specific time period using MATLAB. The time required to generate 50% of the total accumulated NO (*t*_50_) was determined from the cumulative NO-generation profile. The data can also be represented in NO flux units of (× 10^−10^ mol cm^−2^ min^−1^). NO recovery calculations were performed using the theoretical amount of moles released, employing simple dimensional analysis as the basis. The apparent surface area-normalized reaction rate constant (*k*_SA_) was determined to evaluate catalytic stability following prolonged aqueous exposure. The NO-generation profiles were converted to NO flux, and the post-peak decay region of each profile was fitted to a first-order exponential model. The apparent rate constant was normalized to the exposed coating surface. Resulting *k*_SA_ values were compared between freshly prepared and PBS-incubated coatings. Final data for NO generation measurements are reported as the mean ± standard deviation (SD) with *n* ≥ 6 for each sample type unless otherwise stated.

### 2.5. Metal leachates analysis

To quantify metal leaching at a level comparable to the detection limits of both the Cu and Zn quantification kits, one 8 mm film was submerged in a 1.5 mL microcentrifuge tube in 1 mL of PBS for 72 h, shaking at 37 °C. The copper assay detects Cu^2+^*via* chromogenic chelation, producing an absorbance peak at 359 nm, while the zinc assay employs a selective fluorescent probe that increases emission upon Zn^2+^ binding (Ex/Em = 485/525 nm). After 24 h and 72 h, the films were removed, and the remaining PBS was used to determine the relative levels of Cu and Zn in solution.

Using a Cu colorimetric assay kit (sensitivity range of 7 µL dL^−1^ to 300 µL dL^−1^) and a Zn fluorometric assay kit (sensitivity of 13 ng mL^−1^), the quantitative amount of metals identified was recorded. Both assays were run independently but used the same leachate solution. Results were normalized to the total surface area of films submerged in PBS during the incubation period. For detailed *p*-values and statistical significance, please refer to Tables S14 and S15.

### 2.6. Cytocompatibility assessment

Mouse fibroblast cells were revived from cryopreservation and maintained in complete cell culture media (DMEM, 10% FBS, 1% PS) in a 75 cm^2^ tissue-culture treated flask. The cells were maintained at 37 °C with 5% CO_2_. Routine maintenance involved detachment of the adhered cell monolayer using 0.25% (w/v) trypsin containing 0.53 mM EDTA. Following detachment, cells were washed in fresh media *via* centrifugation to remove residual trypsin. The washed cells were then resuspended in fresh media and either seeded into a new flask or plated for experimental use.

#### 2.6.1. Direct-contact cytotoxicity

For experimentation, cells were maintained as described previously. Upon resuspension in fresh complete medium, cell counting was performed using an automatic Eve cell counter (NanoEnTek, Waltham, MA). The cells were then seeded into 24-well tissue culture-treated polystyrene plates at a concentration of 50,000 cells per well. Incubation of the plates at 37 °C with 5% CO_2_ overnight allowed for cell adhesion and growth, reaching approximately 80% confluency.

On the following day, all materials utilized for cytocompatibility testing were UV-sterilized, including the sample films, culture plate inserts, and 8 mL vials for subsequent use. The previous media in the plates was replaced with fresh complete media. Hanging cell culture inserts were then added to each well individually. Sterilized film samples were delicately placed into the inserts to simulate a direct leaching environment. The nature of the well inserts allowed for media to completely submerge the film inside to ensure proper mimicking of the physiological environment. NO generation was initiated by periodic injections of GSNO (1 µM) and GSH (30 µM) every hour for 4 h, with the 1 h interval selected based on the duration of NO release from the substrates in solution.

After 4 h, the study was terminated using an MTT (3-(4,5-dimethylthiazol-2-yl)-2,5-diphenyltetrazolium bromide) colorimetric assay. The films, inserts, and media were removed from each well, and the remaining cell monolayers were supplemented with 0.5 mg mL^−1^ of MTT reagent. This reagent permeated the cells and reacted with any active cellular metabolisms, resulting in the formation of purple formazan crystals over 1–2 h at 37 °C with 5% CO_2_. These crystals were then dissolved using dimethyl sulfoxide (DMSO) to generate a gradient of purple solutions, which were analyzed using a Biotek Cytation™ 5 Cell-Imaging Multi-Mode Reader (Winooski, VT). Final cell viability was determined by comparing absorbance readings from the experimental groups against control wells that were not treated (eqn (4)).4
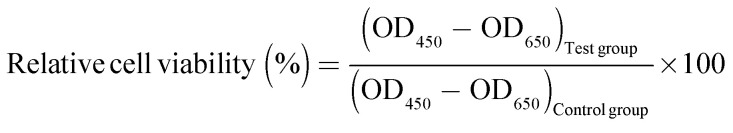


#### 2.6.2. Leachate-based cytotoxicity

To assess cytocompatibility following prolonged exposure to potential leachable species, 24 h material leachates were prepared from control, ZnO and ZnO/10Cu samples. Briefly, sterilized samples were incubated in complete cell culture medium at an extraction ratio of approximately 1 cm^2^ mL^−1^ for 24 h at 37 °C. Following extraction, the leachates were collected and applied directly to 3T3 fibroblasts that had been seeded in 96-well tissue culture-treated polystyrene plates at a density of 5,000 cells per well and cultured overnight to approximately 80% confluency. After 24 h of exposure, relative cell viability was evaluated using the MTT assay as described in Section 2.6.1.

### 2.7. Antibacterial assessment

The combination strategies of the final material aim to reduce planktonic bacterial counts, prevent bacterial adhesion, and induce bacterial killing should adhesion occur.

To quantify antibacterial activity, single colonies of *S. aureus* or *E. coli* were isolated and inoculated into LB media and grown at 37 °C under orbital shaking (150 RPM) until reaching the log phase of growth. The suspension was then centrifuged at 4400 RPM (3000 × *g*) for 7 min (Eppendorf 5702) to yield a bacterial pellet. LB media was decanted, and the bacterial pellet was resuspended in sterile PBS and adjusted to an optical density (OD_600_) of 0.1. Prior to each biological replicate, bacterial enumeration was performed to correlate an OD_600_ of 0.1 with the corresponding bacterial concentration, confirming an inoculum of approximately 10^8^ CFU mL^−1^ before use in antibacterial assays.

#### 2.7.1. Vascular mimicking bacterial adhesion assessment

The following assays assessing both bacterial adhesion and planktonic viability were performed independently using *S. aureus* and *E. coli* under identical experimental conditions. For each adhesion assessment, samples were evaluated under two conditions (*n* = 4 per condition): (i) films without GSNO/GSH [Control (−), ZnO/10Cu (−)] and (ii) corresponding films supplemented with GSNO (1 µM) and GSH (30 µM) [Control (+), ZnO/10Cu (+)], for a total of *n* = 8 per sample type.

Each sample was exposed to 1 mL of 0.1 OD_600_ bacterial solution for a total of 4 h, with respective injections every 1 h (4 injections total). After a 4 h incubation, samples were taken out of the bacterial suspension and rinsed 3 times in sterile PBS to remove any loosely adhered bacteria. Films were then placed in 1 mL of sterile PBS and were homogenized to remove any adhered bacteria. Homogenized solutions were diluted to provide countable colony counts and plated onto LB agar using an Eddy Jet W2 spiral plater (IUL, Farmingdale, NY). Once the dilutions were spiral plated, LB agar Petri dishes were placed upside down and incubated overnight at 37 °C to allow viable colonies to grow. Colonies were counted and subsequently analyzed with a SphereFlash automated colony counter (IUL, Farmingdale, NY). All sample types were plated undiluted (neat) or diluted at 1 : 100 in sterile PBS (2nd dilution).

#### 2.7.2. Vascular mimicking planktonic killing assessment

Similar to adhesion assessments, each film was exposed to 1 mL of 0.1 OD_600_ bacterial solution for 4 h with the identical injection schedule. After 4 h incubation, samples were taken out of the bacterial suspension. The remaining bacterial solutions were diluted to provide countable colony counts and plated onto LB agar and treated identically to adhesion study conditions. All sample types were plated at ratios of 1 : 100 (2nd dilution) or 1 : 10 000 (4th dilution) in sterile PBS. For detailed p-values and statistical significance, please refer to Tables S16 and S17.

### 2.8. Statistical analyses

All data is reported as mean ± standard deviation unless otherwise specified. Statistical significance between sample types was obtained from performing ordinary one-way analysis of variance (ANOVA) tests. Comparisons with a *p*-value of <0.05 were deemed significant. Any significance data not displayed can be found in supplementary information.

## 3. Results and discussion

### 3.1. Synthesis and surface characterization

A lotus leaf-inspired non-adhesive superhydrophobic coating was achieved through the incorporation of silanized ZnO and Cu particles within a siloxane matrix. Non adhesive superhydrophobic performance is defined by static water contact angles (WCA) greater than 150° and water contact angle hysteresis (CAH) below 10°.^12,25–29^ These properties arise from the combined effects of micro- and nanoscale roughness and low-surface-energy chemistry. In this work, hydrophilic metal and metal oxide particles were modified with octadecyltrimethoxysilane (ODTMS), a long-chain silane, to impart low surface energy. The viscosity of the metal/metal-oxide-ODTMS suspension was then adjusted to a paint-like consistency and incorporated within a polydimethylsiloxane (PDMS) base to improve adhesion of the coating to underlying PDMS substrates.

**Fig. 1:**
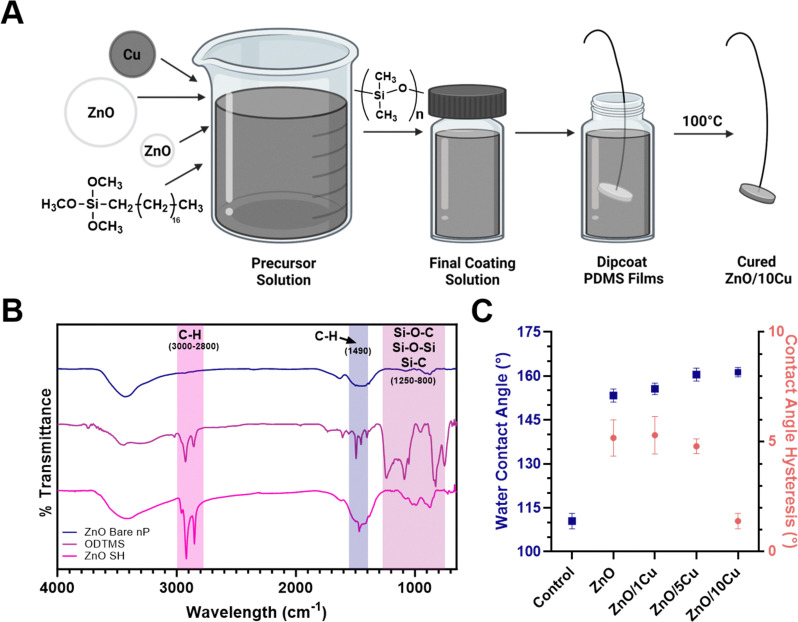
Fabrication and characterization of superhydrophobic samples. (A) Schematic illustration of the fabrication process for ZnO/10Cu, (B) FTIR for untreated ZnO nanoparticles, ODTMS, and ZnO superhydrophobic particles, (C) water contact angle (blue) and contact angle hysteresis (red) measurements; contact angle hysteresis for control PDMS is >20°. *n* ≥ 4; data presented as mean ± SD.

The three-step fabrication process for creating the superhydrophobic, NO-generating surface is illustrated in Fig. 1A. A precursor solution containing ZnO nanoparticles and microparticles, Cu nanoparticles, and ODTMS in hexane was first prepared. The use of ZnO microparticles and nanoparticles, together with intermediate-sized Cu nanoparticles, was intentionally selected to establish a hierarchical distribution of feature sizes across multiple scale lengths. Sylgard-184 PDMS was subsequently incorporated at 10 wt%, and the resulting mixture was applied to PDMS films *via* dip-coating and cured to form superhydrophobic surfaces. Bare Sylgard-184 PDMS films served as controls for performance evaluation.

The silanization of the metal/metal oxide particles by ODTMS was hypothesized to embed superhydrophobic properties. ODTMS, a long-chain aliphatic silane, was selected as a non-fluorinated, low-surface-energy modifier to reduce potential toxicity concerns associated with fluorinated silanes.^30–32^ To verify this modification, unmodified and silanized metallic particles derived from the precursor solution were analyzed by FTIR (Fig. 1B). Modified ZnO particles are denoted as ZnO SH to distinguish them from unmodified ZnO. In addition to spectroscopic confirmation, covalent silanization was further supported by a change in particle wettability from hydrophilic to superhydrophobic (Fig. S2).

Based on the molecular structure of ODTMS, FTIR spectra were expected to exhibit characteristic vibrations associated with C–H stretching, Si–O–C, Si–O–Si, and Si–C bonds. As shown in Fig. 1B, neat ODTMS displayed prominent peaks between 2800–3000 cm^−1^ and near 1490 cm^−1^, corresponding to C–H stretching and bending of the aliphatic chains. Additional peaks near 1250 cm^−1^, 1100 cm^−1^, and within the 800–1000 cm^−1^ region were assigned to Si–O–C, Si–O–Si, and Si–C stretching vibrations, respectively.

Compared to unmodified ZnO, silanized ZnO (ZnO SH) exhibited pronounced peaks in the 2800–3000 cm^−1^ and 1490 cm^−1^ regions, along with increased intensity within the 900–1100 cm^−1^ range, indicating successful grafting of ODTMS onto the particle surface. Collectively, these spectral features confirm effective silanization, which is necessary to impart low surface energy and enable subsequent formation of non-adhesive superhydrophobic coatings.

Hierarchical micro/nanoscale surface architecture has been widely reported as essential for achieving durable superhydrophobicity. Previous studies have demonstrated that micro- or nano-structured surfaces individually fail to exhibit stable superhydrophobic behavior, whereas hierarchical structures enhance wettability by stabilizing the trapped air layer and improving resistance to wetting transitions.^33,34^ Consistent with these principles, varying ratios of ZnO nanoparticles and microparticles were initially explored to assess general coating robustness (Table S1 and Fig. S3). To minimize experimental values and enable direct comparison among formulations, a 50 : 50 ratio of ZnO nanoparticles to microparticles was selected. This ratio was used for all subsequent studies.

**Fig. 2:**
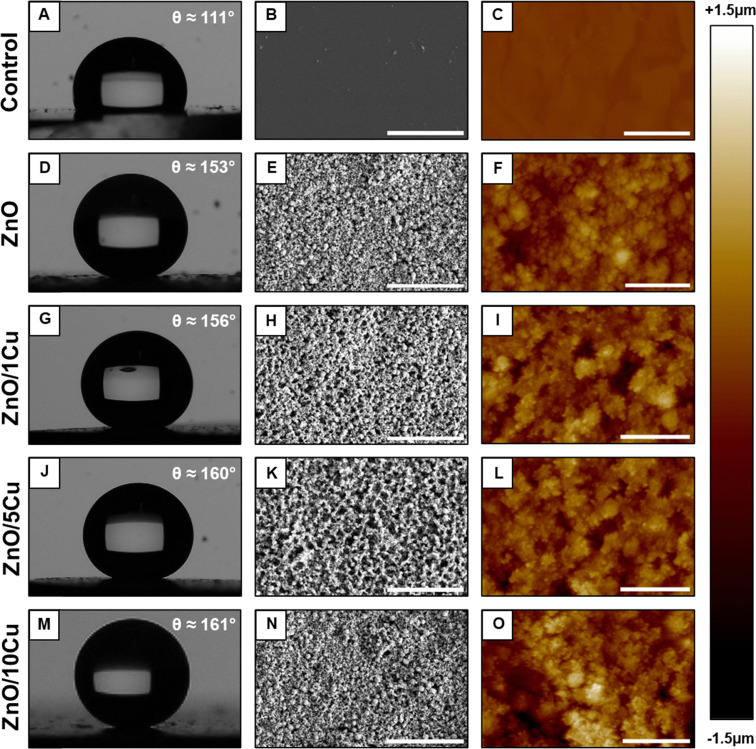
Surface Characterization of ZnO and Cu-Doped Samples. WCA (A), (D), (G), (J) and (M), SEM (B), (E), (H), (K) and (N), and AFM (C), (F), (I), (L) and (O) images characterizing the surface of (A)–(C) control, (D)–(F) ZnO, (G)–(I) ZnO/1Cu, (J)–(L) ZnO/5Cu, and (M–O) ZnO/10Cu samples. SEM scale bars: 20 µm. AFM scale bars: 5 µm. *n* ≥ 4; data presented as mean ± SD.

All fabricated coatings exhibited static water contact angles (WCA) >150°, in contrast to control PDMS, which displayed an average WCA of 111° (Fig. 1C, 2 and Tables S3, S4). In addition, all superhydrophobic samples demonstrated contact angle hysteresis (CAH) values <10°, confirming their non-adhesive superhydrophobic behavior, whereas the control PDMS displayed a substantially higher CAH (>20°), consistent with its conventional hydrophobic character.

As Cu content increased, WCA increased and CAH decreased. This trend is attributed to enhanced hierarchical structuring arising from the introduction of Cu nanoparticles (40–60 nm), which provide intermediate feature sizes between ZnO nanoparticles (10–30 nm) and ZnO microparticles (<5 µm). The distribution of particles across micro- and nanoscale ranges promotes stabilization of the trapped air layer and improves resistance to wetting.

Surface morphology and coating uniformity were further examined by SEM, EDS, and AFM (Fig. 2). All coated samples exhibited textures distinctly different from control PDMS, confirming successful deposition of the particle-based coating. No pronounced morphological differences were observed among ZnO, ZnO/1Cu, ZnO/5Cu, and ZnO/10Cu by SEM. Elemental mapping revealed uniform distributions of Zn and Cu across the coating surface (Fig. S4), with quantitative elemental compositions provided in Table S5. These results indicate consistent incorporation of both metal and metal oxide particles throughout the coating.

AFM analysis revealed a progressive increase in root-mean-square roughness (*R*_q_) with increasing Cu content, from 19.58 ± 5.31 nm for control films to 177.25 ± 23.46 nm for ZnO, 236 ± 22.66 nm for ZnO/1Cu, 288 ± 9.09 nm for ZnO/5Cu, and 353.75 ± 26.04 nm for ZnO/10Cu. All coated samples were significantly rougher than the control (Table S6). The monotonic increase in roughness with Cu incorporation is consistent with the observed improvements in WCA and CAH, further supporting the role of hierarchical surface architecture in governing superhydrophobic performance.

**Fig. 3:**
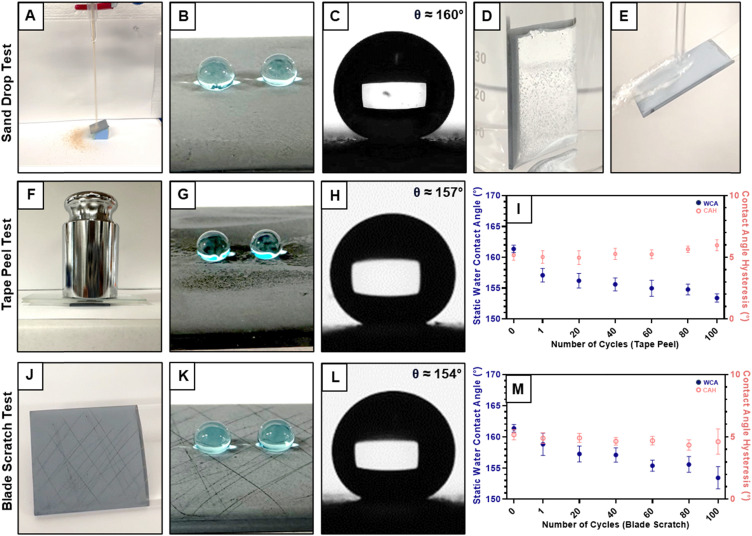
Durability Testing of ZnO/10Cu Surface. (A) Sand drop test set-up, (B) 5 µL droplets of DI water on ZnO/10Cu post-sand drop, (C) water contact angle measurement of ZnO/10Cu post-sand drop, (D) ZnO/10Cu sample submerged in DI water with visible air plastron layer, (E) water-jet test on ZnO/10Cu, (F) tape peel test set-up, (G) 5 µL droplets of DI water on ZnO/10Cu after the first cycle of tape peeling, (H) water contact angle measurement of ZnO/10Cu after the first cycle of tape peeling, (I) ZnO/10Cu performance to multiple cycles of tape-peeling, (J) ZnO/10Cu after first cycle of blade scratching, (K) 5 µL droplets of DI water on ZnO/10Cu after the first cycle of blade scratching, (L) water contact angle measurement of ZnO/10Cu after the first cycle of blade scratching, (M) ZnO/10Cu performance to multiple cycles of blade scratching. *n* ≥ 4; data presented as mean ± SD.

The mechanical durability of the ZnO/10Cu coating was evaluated to assess the stability of the embedded non-adhesive superhydrophobicity under representative mechanical and aqueous stress conditions (Fig. 3). Following a sand-drop test in which 10 g of sand was dropped from a height of 20 cm, the ZnO/10Cu surface maintained a WCA of 159° ± 1.5, indicating minimal disruption of superhydrophobic behavior (Fig. 3A–C and Video S1). When submerged in deionized water, the ZnO/10Cu surface exhibited a visible air plastron layer (Fig. 3D and Video S2), confirming the presence of a trapped air layer within the surface texture. Under continuous water-jet exposure, the coating retained its integrity and water-repellent behavior, with droplets exhibiting pronounced beading and rapid roll-off (Fig. 3E and Video S3), demonstrating resistance to dynamic aqueous shear.

Adhesive tape peeling and blade scratching tests were conducted to further evaluate mechanical robustness. Notably, after 100 cycles of tape peeling, the coating maintained superhydrophobicity, with WCA >150° and CAH <10°, despite a slight decrease in WCA after the initial cycle (Fig. 3F–I and Table S7). Similarly, following repeated blade-scratching (Video S4), WCA remained >150° and CAH remained <10° (Fig. 3J–M and Table S8), indicating that the hierarchical surface structure is preserved even under repeated mechanical disruption.

Collectively, these results demonstrate that the ZnO/10Cu coating exhibits robust mechanical stability, retaining superhydrophobic performance under repeated abrasion, adhesive tape-peeling (>100 cycles), and aqueous exposure. Although a modest decrease in WCA was observed following repeated scratching, the coating maintained high water-repellent behavior, highlighting its resistance to mechanical degradation and addressing a common limitation of mechanically fragile superhydrophobic coatings.

### 3.2. Investigation of nitric oxide generation

**Fig. 4:**
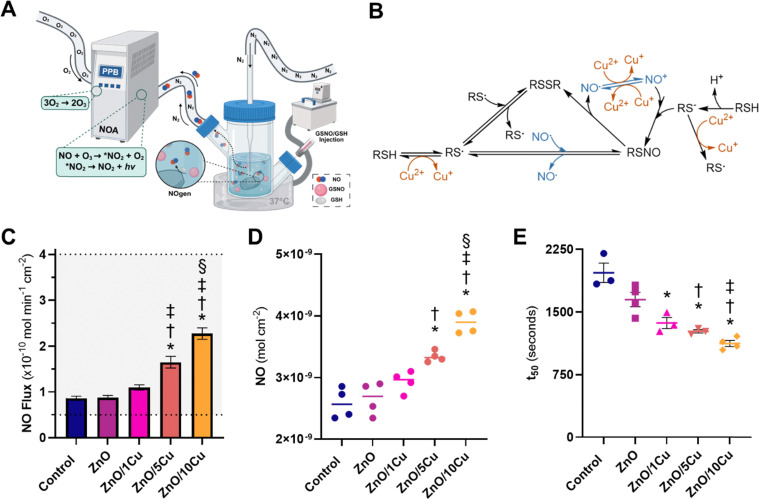
Nitric oxide generation measurements. (A) Schematic and chemiluminescent method for detecting NO generation from materials, (B) schematic for a small selection of interactions between GSNO, GSH, and Cu ions,^17–20^ (C) average peak NO flux (the shaded region represents the reported range of physiological endothelial NO flux, ∼0.5–4 × 10^−10^ mol cm^−2^ min^−1^), (D) accumulated NO normalized to surface area, (E) NO *t*_50_ in response to material. *p < 0.05 *vs*. Control, ^†^*p* < 0.05 *vs*. ZnO, ^‡^*p* < 0.05 *vs*. ZnO/1Cu, ^§^*p* < 0.05 *vs*. ZnO/5Cu. *n* ≥ 6; data presented as mean ± SD.

The ability of the metal-based superhydrophobic coatings to catalytically generate NO from physiological RSNOs was investigated under low-end physiological GSNO/GSH concentrations. All samples, including the control PDMS, were exposed to identical injections of GSNO and GSH to ensure that differences in NO release reflected catalytic activity at the material surface rather than variations in substrate availability (Fig. 4A). Catalytic NO generation is sensitive to the availability of redox-active metal species, making consistent particle loading across samples critical for meaningful comparison of NO-generation performance.

To ensure uniformity, the mass of particles deposited on each substrate was quantified after excluding contributions from the PDMS substrate and solvent (Table S9). No significant differences in particle loading were observed among sample types, indicating that variations in ZnO : Cu ratios did not introduce systematic differences in coating mass. These results enable direct comparison of catalytic activity and NO-generation behavior across formulations.

Catalytic generation of NO from RSNOs by metal-based materials has been widely reported, particularly for copper-containing systems in which Cu^2+^ is reduced to Cu^+^ by thiolates or other physiological reducing agents, followed by Cu^+^-mediated decomposition of GSNO to release NO (Fig. 4B).^18–20^ This Cu-mediated RSNO decomposition pathway has been well established in NO-generating biomaterials, including previous studies from our group demonstrating copper-driven catalytic NO generation from physiological *S*-nitrosothiols.^35,36^ Similar redox-active behavior has also been reported for other transition metals, including iron, nickel, cobalt, manganese, and zinc, depending on oxidation state and coordination environment.^21,37^ In the context of these established mechanisms, the NO-generation performance of the fabricated coatings was systematically evaluated. Three key metrics were examined: average peak NO flux, total accumulated NO normalized to surface area, and *t*_50_, defined as the time required for 50% of the total NO load to be released following exposure to GSNO/GSH.

Average peak NO flux increased with increasing Cu content in the coatings, with ZnO/10Cu exhibiting the highest peak flux (2.28 ± 0.25 × 10^−10^ mol cm^−2^ min^−1^), followed by ZnO/5Cu (1.65 ± 0.22 × 10^−10^ mol cm^−2^ min^−1^), ZnO/1Cu (1.10 ± 0.10 × 10^−10^ mol cm^−2^ min^−1^), and ZnO (0.88 ± 0.09 × 10^−10^ mol cm^−2^ min^−1^), while the control PDMS displayed the lowest NO flux (0.86 ± 0.08 × 10^−10^ mol cm^−2^ min^−1^) (Fig. 4C and Table S10). Peak NO flux primarily reflects the rate at which RSNOs are catalytically decomposed at the material surface. Accordingly, the observed increase in peak flux with Cu incorporation indicates enhanced catalytic activity arising from a greater density of redox-active Cu sites capable of facilitating the Cu^2+^/Cu^+^ redox cycle. Importantly, peak flux alone does not describe the total amount of NO generated, necessitating evaluation of accumulated NO to assess true NO-generation capacity.

Total accumulated NO, normalized to surface area, also increased with Cu content, with ZnO/10Cu generating the highest accumulated NO among all sample types and exhibiting an 86.40 ± 4.0% increase in NO generated relative to the PDMS control (Fig. 4D and Tables S11, S12). In absolute terms, ZnO/10Cu produced 3.90 ± 0.18 nmol cm^−2^ accumulated NO and significantly exceeded ZnO/5Cu (3.34 ± 0.09 nmol cm^−2^). ZnO/5Cu and ZnO/1Cu produced greater accumulated NO than ZnO and the control, while ZnO displayed only a slight, non-significant increase relative to the control. The limited NO generation observed for ZnO alone likely reflects the absence of efficient redox cycling at the ZnO surface. Metal oxides such as ZnO possess relatively stable oxidized metal centers and higher redox potentials than their metallic counterparts, which can limit their participation in catalytic redox cycling.^37,38^ While ZnO can participate in surface interactions with thiols, it lacks the readily accessible redox couple required to efficiently catalyze RSNO decomposition. In contrast, incorporation of Cu introduces redox-active sites capable of cycling between Cu^2+^ and Cu^+^, which facilitates sustained catalytic decomposition of GSNO and subsequent NO release.

To further probe the influence of Cu content, coatings containing Cu concentrations above 10 wt% were examined, yielding a ZnO/20Cu formulation (Fig. S5). However, accumulated NO generated from ZnO/20Cu was not significantly different from that of ZnO/10Cu, indicating that 10 wt% Cu represents a practical upper limit for maximizing NO-generation capacity within this system.

Kinetic persistence of NO generation was evaluated using *t*_50_, defined as the time required for 50% of the total NO to be released following exposure to GSNO/GSH (Fig. 4E and Table S13). The *t*_50_ decreased with increasing Cu content, with ZnO/10Cu exhibiting a *t*_50_ of 1123 ± 70 s compared to 1970 ± 200 s for the control. This reduction in *t*_50_ further supports a Cu-mediated catalytic mechanism, in which higher densities of redox-active sites accelerate RSNO decomposition and NO release.

To assess the stability of catalytic NO-generation following prolonged aqueous exposure, ZnO/10Cu samples were immersed in PBS at 37 °C for 72 h prior to reevaluation of their catalytic performance (Fig. S6). Following incubation, the coating retained a highly hydrophobic surface, exhibiting a WCA of 143°, while catalytic functionality was maintained. Interestingly, the apparent surface area-normalized reaction rate constant (*k*_SA_) increased significantly following PBS incubation relative to freshly prepared coatings, indicating that extended aqueous exposure did not impair the catalytic activity of the coating. These findings are consistent with the catalytic functionality remaining associated with the coating following prolonged PBS incubation and further support the stability of the NO-generating surface under physiologically relevant aqueous conditions.

Importantly, NO-generation was evaluated under low, physiologically relevant RSNO concentrations (1 µM GSNO, 30 µM GSH), rather than the elevated RSNO concentrations often used in mechanistic studies or systems that rely on finite NO donor reservoirs. The peak NO flux observed for all samples following exposure to GSNO/GSH falls within the range of reported endothelial NO flux values (∼0.5–4 × 10^−10^ mol cm^−2^ min^−1^),^39^ indicating that NO generation across all formulations occurs within a physiologically relevant regime. Notably, ZnO/10Cu exhibits the highest flux within this range, reflecting enhanced catalytic activity while remaining within biologically relevant limits. While the total accumulated NO is lower than that achieved in donor-loaded systems, it reflects the limited availability of endogenous NO reservoirs under physiological conditions. Critically, NO-generation in this system is localized at the material interface, where even modest fluxes are sufficient to drive biological responses. This is particularly notable given that NO-generation is achieved through a surface coating alone, without reliance on elevated donor concentrations in solution or incorporation of NO donors within the bulk material.

Collectively, these results demonstrate that Cu incorporation not only accelerates NO-generation kinetics but also increases overall NO yield. The distinction between increased peak flux (reflecting catalytic activity) and increased accumulated NO (reflecting total generation capacity) confirms that ZnO/10Cu functions as an effective NO-generating surface. With ZnO/10Cu exhibiting the highest peak flux, the highest accumulated NO, and the shortest *t*_50_ among the tested formulations, this composition was selected for subsequent biological studies. However, given the potential toxicity of metal-containing materials, comprehensive metal-leaching studies and cytocompatibility assessments were performed prior to antibacterial evaluation.

### 3.3. Evaluation of metal leaching

Given the incorporation of metal and metal oxide particles within the coating, quantitative assessment of Zn and Cu leaching was performed to evaluate material stability and potential toxicity. Samples were incubated in PBS for 72 h under conditions described in the Methods, after which Zn and Cu concentrations in the leachates were quantified using metal detection kits.

Low levels of Zn and Cu were detected for all sample types (Table 2 and Tables S14, S15) ZnO exhibited minimal Zn release, and incorporation of Cu did not increase Zn leaching relative to ZnO alone. For Cu-containing formulations, measurable Cu leaching was observed; however, the detected concentrations were substantially below toxic levels reported in literature.^40^ As expected, the mass of leached Cu increased with increasing Cu content, though the percentage of Cu released relative to the amount deposited onto each film remained below 0.2% for all formulations.

**Table 2:** Zinc and copper metal leaching

Formulation	Cu leachates (µg cm^−2^)	Zn leachates (µg cm^−2^)	Cu leached^a^ (%)	Zn leached^a^ (%)
24 h				
ZnO	—	0.081 ± 0.011	—	0.0017 ± 0.0003
ZnO/1Cu	0.133 ± 0.027	0.059 ± 0.009	0.027 ± 0.057	0.0012 ± 0.0005
ZnO/5Cu	0.191 ± 0.014	0.061 ± 0.011	0.039 ± 0.032	0.0013 ± 0.0002
ZnO/10Cu	0.426 ± 0.009	0.062 ± 0.005	0.087 ± 0.015	0.0013 ± 0.0004
72 h				
ZnO	—	0.137 ± 0.009	—	0.0028 ± 0.0003
ZnO/1Cu	0.263 ± 0.031	0.112 ± 0.011	0.054 ± 0.050	0.0023 ± 0.0003
ZnO/5Cu	0.421 ± 0.045	0.115 ± 0.015	0.086 ± 0.033	0.0024 ± 0.0002
ZnO/10Cu	0.916 ± 0.075	0.114 ± 0.012	0.187 ± 0.015	0.0024 ± 0.0002

a Relative to the amount of Cu and Zn in the coating solutions and the amount deposited onto each film.

These results indicate strong retention of both Zn and Cu within the coating and suggest that NO-generation behavior arises primarily from surface-mediated catalytic activity rather than passive metal ion release. The low extent of metal leaching further supports the suitability of these coatings for subsequent biological evaluation.

### 3.4. Cytocompatibility assessment

**Fig. 5:**
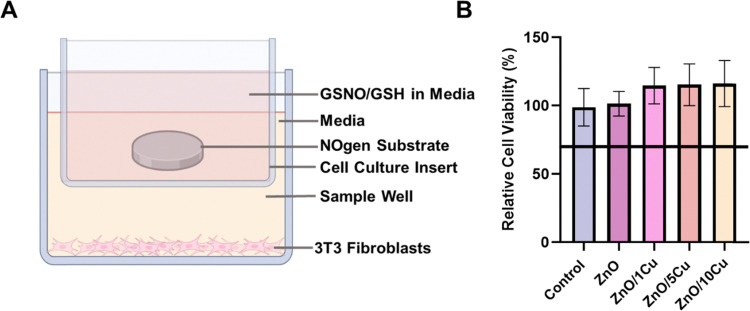
NO-generating cell viability assay and relative cell viability analysis. (A) Schematic of NO-generating cell viability assay, (B) relative cell viability for injected samples with labeled 70% ISO standard. *n* ≥ 4; data presented as mean ± SD.

Cytocompatibility of the fabricated coatings was evaluated using a direct-leaching model with 3T3 mouse fibroblasts. Cells were seeded and cultured to approximately 80% confluency, after which hanging cell culture inserts were placed into each well, and samples were introduced within the inserts to simulate a direct exposure environment (Fig. 5A). To establish NO-generating conditions, GSNO and GSH were injected into each well at final concentrations of 1 µM and 30 µM, respectively, with injections repeated hourly over the 4 h experiment.

Following 4 h of exposure, relative cell viability was quantified using an MTT assay and compared to cells exposed to inserts without material or GSNO/GSH treatment. All sample types exhibited cell viabilities greater than 70%, meeting ISO cytocompatibility criteria (Fig. 5B).^41^ To further assess cytocompatibility under prolonged exposure conditions, 3T3 mouse fibroblast cells were exposed to 24 h leachates from Control PDMS, ZnO, and ZnO/10Cu sample types (Fig. S7). ZnO and ZnO/10Cu were selected to represent the Cu-free and highest Cu-loading formulations, respectively. All groups maintained relative cell viabilities above 90%, exceeding the ISO 10993-5 cytocompatibility threshold of 70%.^41^

These results, in conjunction with the low levels of Zn and Cu leaching, demonstrate that the coatings maintain cytocompatibility while supporting catalytic NO generation under physiologically relevant conditions. Importantly, the preservation of fibroblast viability suggests that NO produced at these low-end physiological RSNO/GSH concentrations does not induce acute cytotoxicity. Notably, the transwell-based exposure model was intentionally designed to assess cytocompatibility of the coating under vascular-mimicking NO-generating conditions, in which endogenous RSNOs are continuously converted to NO at the material interface. Together, these findings indicate that incorporation of redox-active metals within the superhydrophobic matrix enables functional NO generation without compromising cellular compatibility, supporting the suitability of these coatings for subsequent antibacterial evaluation and potential biomedical applications.

### 3.5. Antibacterial assessment

Antibacterial performance of the optimized ZnO/10Cu coating was evaluated using a purpose-designed, vascular-mimicking NO-generating model that integrates physiological RSNO/GSH delivery with surface-mediated catalytic NO-generation. This model was developed to assess antibacterial behavior under conditions that more closely approximate physiologically relevant NO-rich microenvironments. To establish low-end physiological NO-generating conditions, GSNO and GSH were injected hourly to achieve final concentrations of 1 µM and 30 µM, respectively. PDMS, ZnO, and ZnO/10Cu samples were evaluated under untreated (PBS only) and treated (GSNO/GSH) conditions to decouple the effects of substrate chemistry from those of the NO-generating environment. In this system, ZnO provides superhydrophobic surface roughness and modest antibacterial behavior, whereas incorporation of Cu enables catalytic NO generation from physiological levels of RSNOs.

The ZnO/10Cu coating exhibits both non-adhesive superhydrophobicity and catalytic NO-generation; therefore, antibacterial performance was evaluated in terms of (i) bacterial addition to the surface and (ii) planktonic bacterial viability in the surrounding medium. Evaluation of planktonic bacteria is particularly important, as many superhydrophobic surfaces primarily report reductions in surface-associated bacteria rather than true bactericidal activity. Quantifying planktonic bacteria enables assessment of whether additional antimicrobial effects arise from surface-mediated NO generation beyond any intrinsic antibacterial contributions of GSNO/GSH alone.

**Fig. 6:**
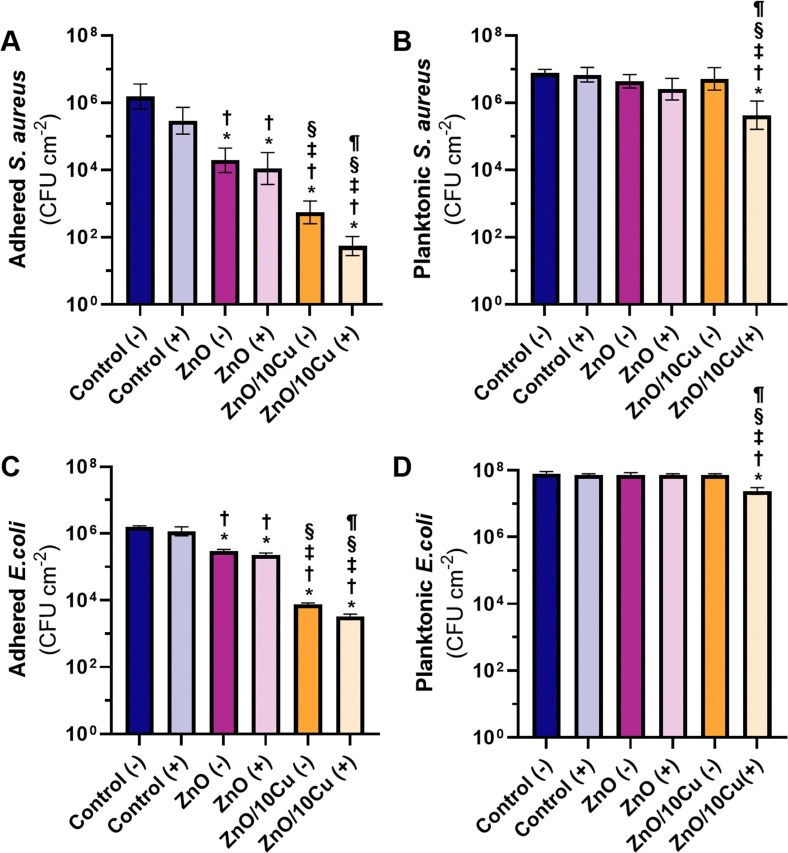
Impact of NO-generating conditions on *S. aureus* and *E. coli* bacterial adhesion and planktonic growth. (A) Adhered log *S. aureus*, (B) planktonic log *S. aureus*. (C) Adhered log *E. coli*, (D) planktonic log *E. coli*. A (+) indicates the presence of physiological levels of GSNO and GSH, and a (−) indicates the absence. *p < 0.05 *vs*. Control (−), ^†^*p* < 0.05 *vs.* Control (+), ^‡^*p* < 0.05 *vs*. ZnO (−), ^§^*p* < 0.05 *vs*. ZnO (+), ^¶^*p* < 0.05 *vs*. ZnO/10Cu (−). *n* ≥ 4; data presented as mean ± SD.

ZnO/10Cu surfaces exhibited significantly fewer adhered *S. aureus* compared to both PDMS and ZnO controls under both untreated and GSNO/GSH-treated conditions (Fig. 6A). Relative to untreated PDMS, ZnO/10Cu demonstrated a 3.45-log reduction in adhered bacteria, while a 4.45-log reduction was observed under NO-generating conditions (Tables S16 and S17), indicating strong antibacterial activity at the material interface. ZnO surfaces also showed reduced bacterial adhesion compared to PDMS, consistent with the anti-adhesive behavior of superhydrophobic surfaces and the modest antibacterial activity reported for ZnO-containing coatings; however, adhered bacterial counts on ZnO remained significantly higher than those observed for ZnO/10Cu.

Only minor, non-significant differences were observed between untreated and GSNO/GSH-treated PDMS and ZnO samples, indicating minimal antibacterial contribution from GSNO/GSH alone at these concentrations. In contrast, a statistically significant ∼1.00-log reduction was observed between untreated and treated ZnO/10Cu samples. These results demonstrate that non-adhesive superhydrophobicity is the dominant contributor to reduced bacterial attachment, while surface-mediated NO-generation provides an additional antibacterial effect even under low, physiologically relevant GSNO/GSH concentrations.

Planktonic *S. aureus* viability was quantified following 4 h exposure to evaluate whether surface-mediated NO-generation impacts bacteria in the surrounding medium in addition to reducing surface adhesion (Fig. 6B). No significant differences in planktonic bacterial counts were observed among untreated PDMS, treated PDMS, untreated ZnO, treated ZnO, or untreated ZnO/10Cu samples. In particular, the absence of antibacterial activity for untreated ZnO/10Cu indicates that the coating itself—including any contributions from residual metal ions or surface-mediated effects in the absence of NO donor chemistry—did not reduce planktonic bacterial viability. In contrast, ZnO/10Cu under NO-generating conditions exhibited a significant reduction in planktonic bacteria, corresponding to approximately a 1.20-log decrease relative to untreated PDMS.

Notably, despite operating under low, physiologically relevant RSNO concentrations, the system achieved substantial antibacterial activity, with multi-log reductions in surface-adhered bacteria and measurable reductions in planktonic populations. This behavior is consistent with a primarily interfacial mechanism, in which localized NO generation at the coating surface drives bacterial inactivation, rather than reliance on bulk-phase antimicrobial release.

These results demonstrate that neither GSNO/GSH alone nor the ZnO/10Cu coating in the absence of NO donor chemistry produced significant planktonic bactericidal activity. Accordingly, antibacterial effects arising from residual metal ion activity or surface-mediated interactions alone can be excluded under the experimental conditions. Rather, the significant planktonic killing observed exclusively for ZnO/10Cu under NO-generating conditions provides direct functional evidence of catalytic NO-generation, confirming that

NO produced at the material interface diffuses into the surrounding medium and exerts antibacterial effects. This represents a critical validation of true NO-generating behavior, extending beyond chemical detection alone to demonstrate biological efficacy.

To further evaluate the antibacterial performance of the coatings against Gram-negative bacteria, assessments of adhered and planktonic *E. coli* were also performed under identical vascular-mimicking NO-generating conditions (Fig. 6C and D). Comparable overall antibacterial trends were observed for *E. coli*, although the magnitude of the antibacterial response was lower than that observed for *S. aureus*. ZnO surfaces also significantly reduced adhered *E. coli* relative to PDMS, although the magnitude of reduction was substantially smaller than that observed for ZnO/10Cu. ZnO/10Cu produced 2.31- and 2.68-log reductions under untreated and NO-generating conditions, respectively, relative to untreated PDMS (Tables S18 and S19). Consistent with the *S. aureus* studies, GSNO/GSH alone did not significantly reduce bacterial adhesion, whereas incorporation of Cu provided the greatest antibacterial benefit. Likewise, no significant reductions in planktonic *E. coli* were observed for untreated PDMS, treated PDMS, untreated ZnO, treated ZnO, or untreated ZnO/10Cu. In contrast, ZnO/10Cu under NO-generating conditions significantly reduced planktonic bacterial counts by 0.52-log relative to untreated PDMS. The reduced susceptibility of *E. coli* is consistent with the presence of a Gram-negative outer membrane, which provides an additional permeability barrier, as well as the well-established nitrosative stress defense mechanisms present in *E. coli*.^42,43^

Collectively, these findings demonstrate that the ZnO/10Cu coating integrates non-adhesive superhydrophobicity with catalytic NO-generation to achieve robust antibacterial performance against both representative Gram-positive (*S. aureus*) and Gram-negative (*E. coli*) bacteria. Superhydrophobic surface features substantially limit initial bacterial attachment, whereas surface-mediated NO-generation provides additional suppression of both adhered and planktonic bacteria under vascular-mimicking conditions. The ability to simultaneously reduce surface-associated bacteria and impact microorganisms in the surrounding medium highlights the advantage of combining passive antibiofouling with localized, catalytic antimicrobial functionality within a single interfacial platform.

## 4. Conclusion

A metal-siloxane-derived, non-adhesive superhydrophobic coating capable of catalytic nitric oxide (NO) generation from physiological *S*-nitrosothiols was developed and systematically evaluated. Unlike prior approaches that rely on cytotoxic fluorosilanes, this platform utilizes ZnO and Cu particles with ODTMS to achieve hierarchical micro/nanoscale roughness, low surface energy, and mechanically robust superhydrophobicity. Incorporation of Cu enables surface-mediated NO generation under low, physiologically relevant RSNO/GSH conditions. Kinetic and cumulative NO analyses confirmed that increasing Cu content enhances both NO-generation rate and total NO output, with ZnO/10Cu identified as the optimized formulation. Notably, NO-generation occurs within the range of endothelial NO flux despite the absence of an embedded donor reservoir, highlighting the system's ability to achieve biologically relevant NO delivery through catalytic, interfacial mechanisms.

Low levels of Zn and Cu leaching, combined with cell viability (>70%), demonstrate that functional performance is achieved without compromising cytocompatibility. Using a vascular-mimicking NO-generating model, the optimized coating substantially reduced *S. aureus* bacterial adhesion (99.99%) and significantly decreased planktonic *S. aureus* viability (89.43%). Comparable overall antibacterial trends were also observed against the representative Gram-negative bacteria *E. coli*, with the optimized coating producing 99.79% and 69.8% reductions in adhered and planktonic bacteria, respectively. These results represent one of the first demonstrations of successful antibacterial performance achieved through catalytic NO-generation from endogenous RSNOs. These results provide direct biological validation of true catalytic NO-generating behavior, extending beyond chemical detection to demonstrate functional efficacy.

Collectively, this work establishes the first integration of non-adhesive superhydrophobicity with catalytic NO generation within a single coating platform. By combining passive antifouling with localized, sustained NO production from endogenous reservoirs—without reliance on elevated donor concentrations or bulk donor incorporation—this approach addresses key limitations of existing antimicrobial and antifouling technologies. This strategy provides a scalable, versatile framework for designing next-generation antibiofouling and antimicrobial surfaces with improved biological relevance and translational potential. Importantly, these findings demonstrate that effective antimicrobial performance can be achieved under low, physiologically relevant NO-generating conditions, emphasizing the need to evaluate NO-generating materials within realistic biological regimes rather than relying on elevated donor concentrations that may overestimate performance.

## Author contributions

Annalise Tucker: conceptualization, methodology, formal analysis, investigation, data curation, visualization, writing – original draft, writing – review & editing. Ekrem Ozkan: conceptualization, methodology, investigation, writing – review & editing. Sarah Wilson: methodology, investigation, data curation, writing – review & editing. Arpita Shome: investigation, data curation, writing – review & editing. Hitesh Handa: conceptualization, visualization, investigation, data curation, supervision, validation, funding acquisition, writing – review & editing, project administration. Elizabeth J. Brisbois: conceptualization, visualization, investigation, data curation, supervision, validation, funding acquisition, writing – review & editing, project administration.

## Conflicts of interest

The authors declare the following competing financial interest(s): Hitesh Handa and Elizabeth J. Brisbois are cofounders and maintain a financial interest in a startup company investigating nitric oxide as a biomedical therapeutic for medical devices.

## Supplementary Material

TB-014-D6TB01036A-s001

TB-014-D6TB01036A-s002

TB-014-D6TB01036A-s003

TB-014-D6TB01036A-s004

TB-014-D6TB01036A-s005

## Data Availability

The data supporting this article has been included as part of the supplementary information (SI). The supplementary information includes additional experimental methods, supporting figures and tables, surface characterization data, nitric oxide generation analyses, antibacterial and cytocompatibility data, durability testing results, particle loading calculations, and supplementary equations that support the findings of this study. See DOI: https://doi.org/10.1039/d6tb01036a.
