# Supplementary material for: Metal-siloxanes derived bio-inspired superhydrophobicity and nitric oxide generation for anti-biofouling clinical applications
Source: J Mater Chem B. 2026 Jul 23. Online ahead of print. doi: 10.1039/d6tb01036a (PMC13422251; doi:10.1039/d6tb01036a)
Supplement: TB-OLF-D6TB01036A-s001 [file TB-OLF-D6TB01036A-s001.pdf]

Supplementary Information (SI) for Journal of Materials Chemistry B.
This journal is © The Royal Society of Chemistry 2026

Metal-Siloxanes Derived Bio-inspired Superhydrophobicity and Nitric Oxide Generation for Antibiofouling Clinical Applications: Supplementary Information

Annalise D. M. Tucker[1], *Ekrem Ozkan*[1], *Sarah N. Wilson*[1], *Arpita Shome*[1], *Hitesh Handa*[1,2], *and Elizabeth J. Brisbois*[1,*]

[1] School of Chemical, Materials and Biomedical Engineering, College of Engineering, University of Georgia, Athens, Georgia 30602, United States

[2] Department of Pharmaceutical and Biomedical Sciences, College of Pharmacy, University of Georgia, Athens, Georgia 30602, United States

*Corresponding author

Dr. Elizabeth J. Brisbois

Associate Professor

University of Georgia

302 East Campus Rd.

Athens, GA 30602

Telephone: 706-542-1243

E-mail: ejbrisbois@uga.edu

Video S1: Video depicting the sand-drop test on ZnO/10Cu.

Video S2: Video depicting the air-plastron layer when ZnO/10Cu is dipped in deionized water.

Video S3: Video depicting ZnO/10Cu subjected to the water-jet test.

Video S4: Video depicting the blade-scratch test on ZnO/10Cu.

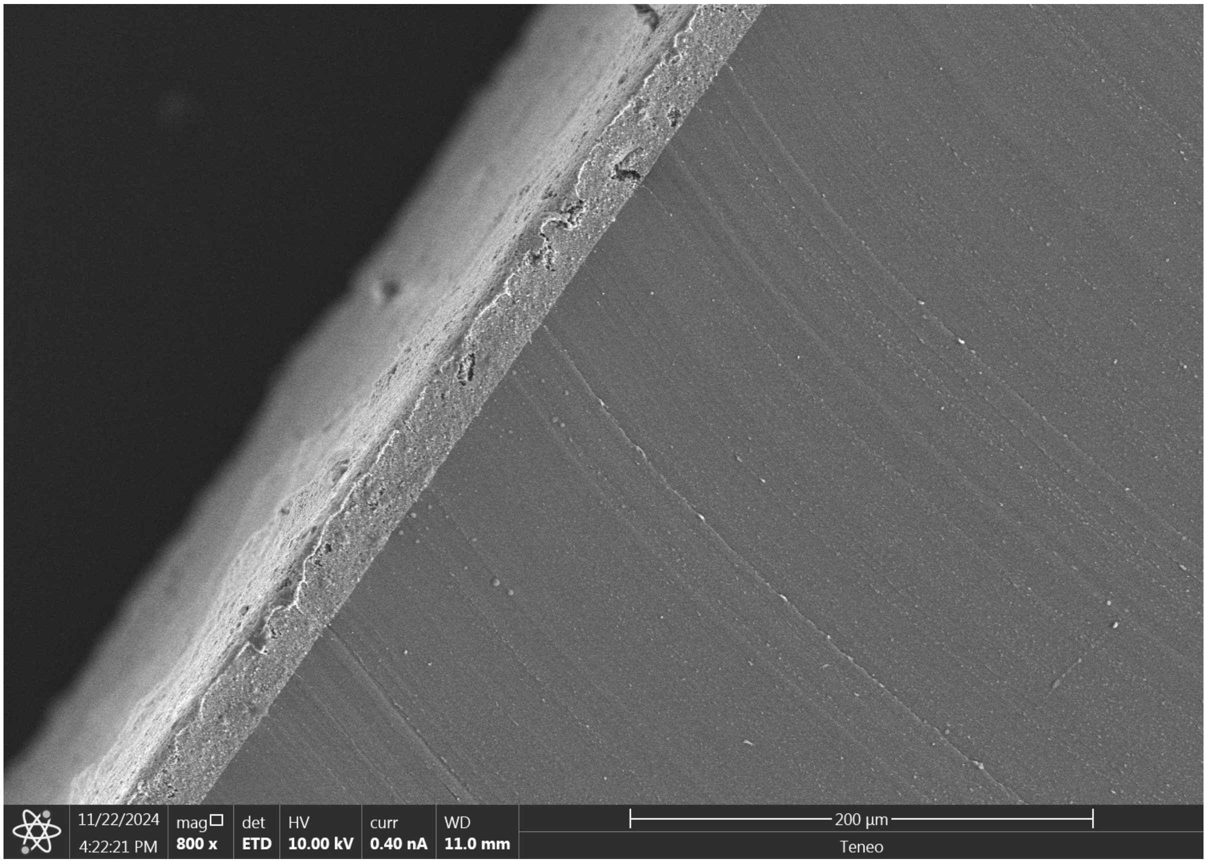

Figure S1. SEM cross-section of ZnO/10Cu. Analyzed coating thickness of 24.45 µm ± 1.04. Measurements of $n \geq 4$; data presented as mean ± SD.

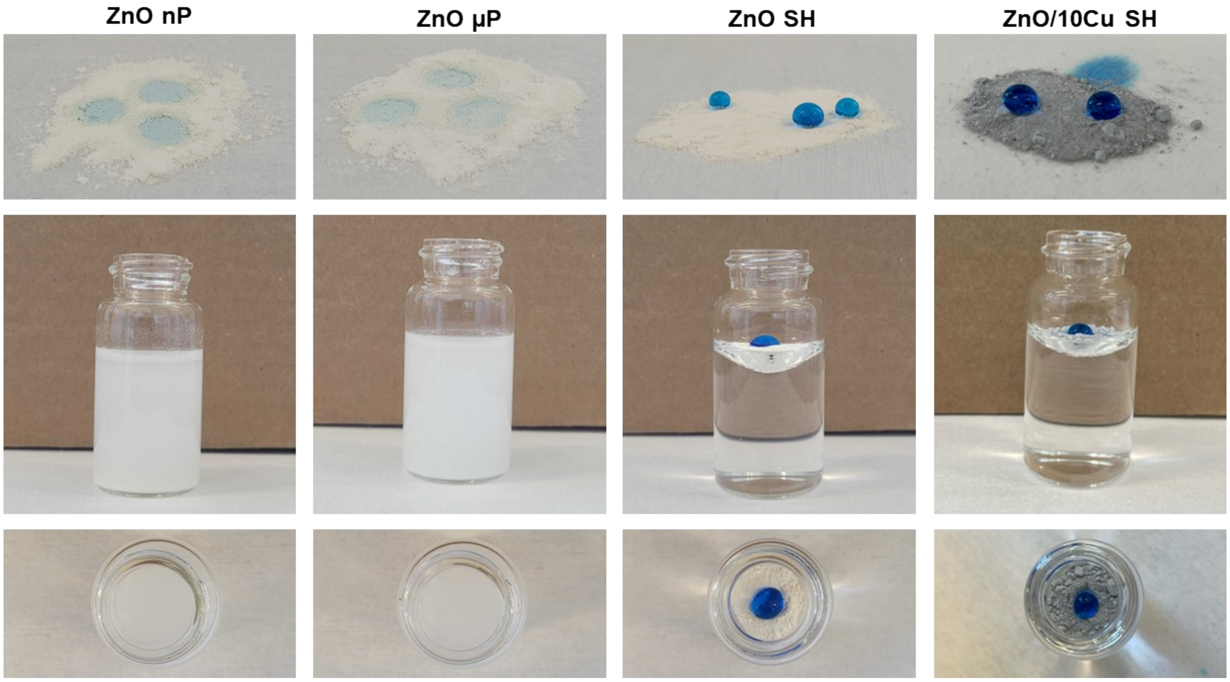

Figure S2. Representation of Successful Silanization of Particles. Neat ZnO nanoparticles, neat ZnO microparticles, superhydrophobic ZnO (ZnO SH), and superhydrophobic ZnO/10Cu (ZnO/10Cu SH) particles exposed to a-d) DI water droplets dyed with methylene blue for color, e-h) particles mixed with DI water. Both neat ZnO particles suspended quickly in the DI water, while superhydrophobic ZnO and ZnO/10Cu particles float on top of the DI water. When a water droplet is added to the top of the floating powder, the droplet remains on top of the particle.

Table S1. Nanoparticle and Microparticle Ratio for Coating Optimization.

Formulation	n-hexane (g)	ODTMS (g)	ZnO µp (g)	ZnO np (g)	DI (µL)
**ZnO 0:100**	20	0.2	0	12	10
**ZnO 25:75**	20	0.2	3	9	10
**ZnO 75:25**	20	0.2	9	3	10
**ZnO 100:0**	20	0.2	12	0	10
**ZnO (50:50)**	20	0.2	6	6	10

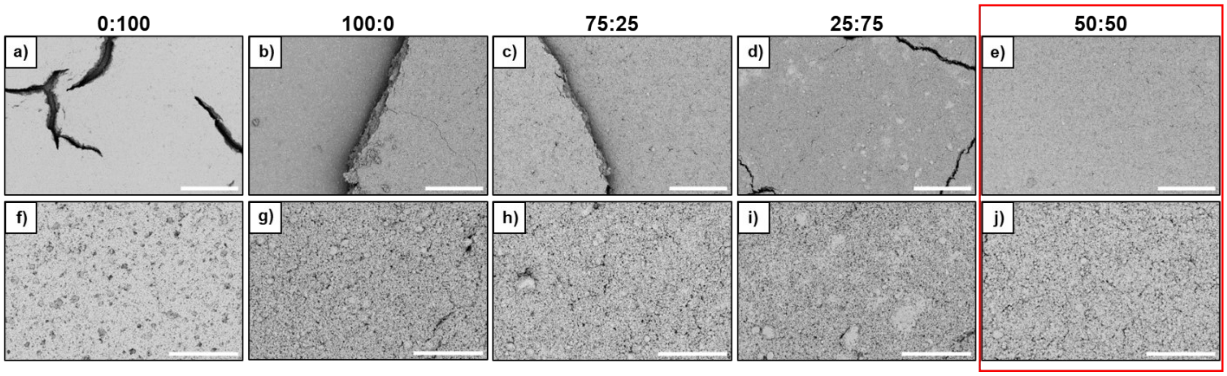

Figure S3. Optimizing nano to microparticle ratio. Severe cracking and shattering of surface coating can be seen in 100:0 and 75:25 samples. Less severe cracking is seen with 0:100 sample, but a solely microstructure surface is not suggested[1]. Minimal cracking can be seen with 25:75, but at the fixed 10wt% PDMS concentration, the 50:50 sample appears to be more robust than the 25:75. Scale bars represent **a-e)** 100µm and **f-j)** 30µm.

Table S3. Statistical Significance (p-values) for Water Contact Angle (WCA).

Formulation	Control	ZnO	ZnO/1Cu	ZnO/5Cu	ZnO/10Cu
**Control**		<0.0001	<0.0001	<0.0001	<0.0001
**ZnO**			0.0960	<0.0001	<0.0001
**ZnO/1Cu**				<0.0001	<0.0001
**ZnO/5Cu**					0.7989
**ZnO/10Cu**					

Table S4. Statistical Significance (p-values) for Contact Angle Hysteresis (CAH).

Formulation	ZnO	ZnO/1Cu	ZnO/5Cu	ZnO/10Cu
**ZnO**		0.9937	0.8476	0.0001
**ZnO/1Cu**			0.7138	<0.0001
**ZnO/5Cu**				0.0004
**ZnO/10Cu**				

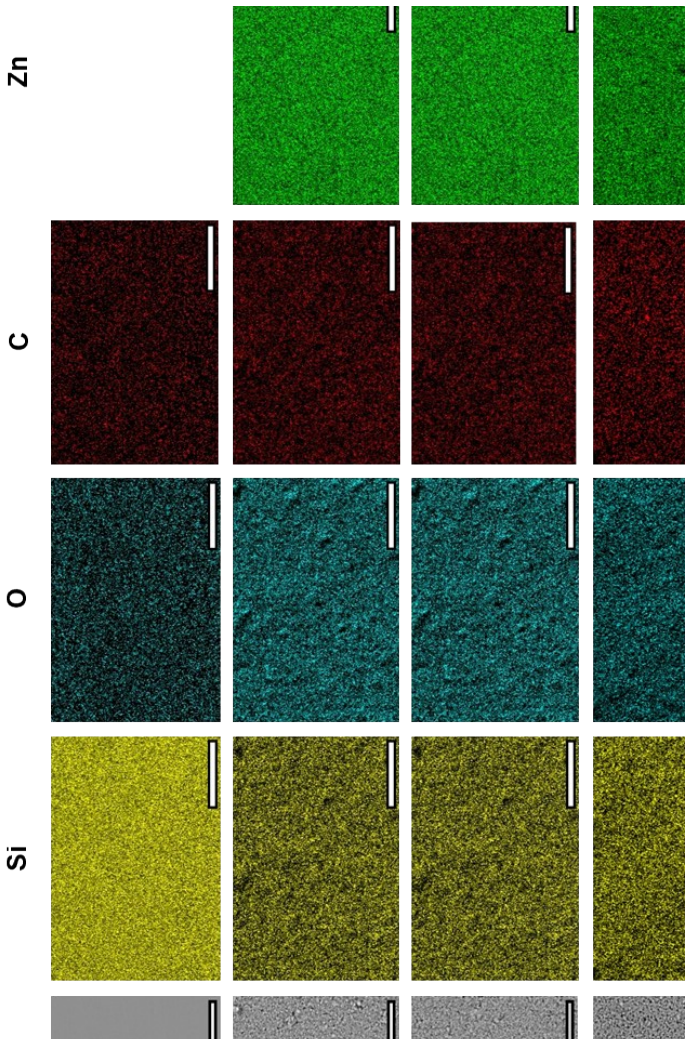

Figure S4. EDS images showing elemental distribution across all sample types. Uniform distribution of silicon (Si), oxygen (O), carbon (C), zinc (Zn), and copper (Cu) in respective samples indicates consistent coating distribution. Scale bars represent 500 µm.

Table S5. Elemental Percentages from EDS Analysis.

Formulation	Si (%)	O (%)	C (%)	Zn (%)	Cu (%)
**Control**	35.2	24.5	40.4	-	-
**ZnO**	6.2	21.5	24.8	47.5	-
**ZnO/1Cu**	6.6	21.1	23.6	48.4	0.3
**ZnO/5Cu**	6.7	20.4	23.6	47.8	1.6
**ZnO/10Cu**	6.5	20.8	23.8	46.0	2.9

Table S6. Statistical Significance (p-values) for Root Mean Square Roughness (Rq).

Formulation	Control	ZnO	ZnO/1Cu	ZnO/5Cu	ZnO/10Cu
**Control**		<0.0001	<0.0001	<0.0001	<0.0001
**ZnO**			0.0047	<0.0001	<0.0001
**ZnO/1Cu**				0.0123	<0.0001
**ZnO/5Cu**					0.0018

**ZnO/10Cu**					

Table S7. Statistical Significance (p-values) for Tape Durability Testing WCA and CAH.

**Water Contact Angle**							
**# of Cycles**	**0**	**1**	**20**	**40**	**60**	**80**	**100**
**0**		0.0557	0.0103	0.0030	0.0007	0.0005	<0.0001
**1**			0.9955	0.9405	0.7449	0.6639	0.1442
**20**				0.9996	0.9756	0.9513	0.4371
**40**					0.9993	0.9970	0.7025
**60**						>0.9999	0.9200
**80**							0.9553
**100**							
**Hysteresis**							
**# of Cycles**	**0**	**1**	**20**	**40**	**60**	**80**	**100**
**0**		>0.9999	0.9999	>0.9999	>0.9999	0.9869	0.8552
**1**			>0.9999	0.9996	0.9998	0.9478	0.7283
**20**				0.9989	0.9993	0.9264	0.6815
**40**					>0.9999	0.9961	0.9139
**60**						0.9944	0.9002
**80**							0.9982
**100**							

Table S8. Statistical Significance (p-values) for Blade Scratching Durability Testing WCA and CAH.

**Water Contact Angle**							
**# of Cycles**	**0**	**1**	**20**	**40**	**60**	**80**	**100**
**0**		0.8193	0.3183	0.2475	0.0365	0.0480	0.0018
**1**			0.9804	0.9678	0.5305	0.6001	0.0806
**20**				>0.9999	0.9503	0.9711	0.4009
**40**					0.9678	0.9827	0.4538
**60**						>0.9999	0.9421
**80**							0.9102
**100**							
**Hysteresis**							
**# of Cycles**	**0**	**1**	**20**	**40**	**60**	**80**	**100**
**0**		0.9997	0.9998	0.9898	0.9940	0.9128	0.9871
**1**			>0.9999	0.9998	>0.9999	0.9876	0.9997
**20**				0.9997	>0.9999	0.9844	0.9996
**40**					>0.9999	0.9996	>0.9999
**60**						0.9989	>0.9999
**80**							0.9997
**100**							

Table S9. Weight of Particles Deposited onto Films. Assuming full evaporation of the solvent and theoretical mass percent of PDMS in the coating to be 10%, 90% of the difference in weight is assumed to be metal nanoparticles and microparticles (**Equation S1**). Final data for overall coating weight is reported as the mean ± standard deviation (SD) with n≥9 for each sample type.

Formulation	Particles Deposited (mg cm^{-2})
**ZnO**	6.48 ± 0.93
**ZnO/1Cu**	6.44 ± 1.26
**ZnO/5Cu**	6.45 ± 1.01
**ZnO/10Cu**	6.47 ± 0.74

$$Mass_{particles}\,(mg) = \left[Mass_{post\ cure}\,(mg) - Mass_{base\ PDMS}\,(mg)\right] * 0.90 \quad \textbf{(Eq.S1)}$$

Table S10. Statistical Significance (p-values) for NO Flux.

Formulation	Control	ZnO	ZnO/1Cu	ZnO/5Cu	ZnO/10Cu
**Control**		0.9990	0.1675	<0.0001	<0.0001
**ZnO**			0.2437	<0.0001	<0.0001
**ZnO/1Cu**				0.0010	<0.0001
**ZnO/5Cu**					0.0003
**ZnO/10Cu**					

Table S11. NO Generation Analysis. NO Recovery is relative to the theoretical amount of NO release from 1μM GSNO in ideal situations. An increase in NO recovery indicates the release of more moles of NO in response to reducing agent GSH and metal interactions with GSNO.

Formulation	NO (nmol)	NO Recovery (%)
**Control**	3.43 ± 0.33	57.24 ± 5.53
**ZnO**	3.53 ± 0.36	58.91 ± 5.96
**ZnO/1Cu**	3.90 ± 0.23	65.02 ± 3.81
**ZnO/5Cu**	4.44 ± 0.12	74.04 ± 1.96
**ZnO/10Cu**	5.18 ± 0.24	86.40 ± 4.01

Table S12. Statistical Significance (p-values) for Accumulated NO.

Formulation	Control	ZnO	ZnO/1Cu	ZnO/5Cu	ZnO/10Cu
**Control**		0.9879	0.2309	0.0031	<0.0001
**ZnO**			0.4387	0.0069	<0.0001
**ZnO/1Cu**				0.1340	0.0004
**ZnO/5Cu**					0.0269
**ZnO/10Cu**					

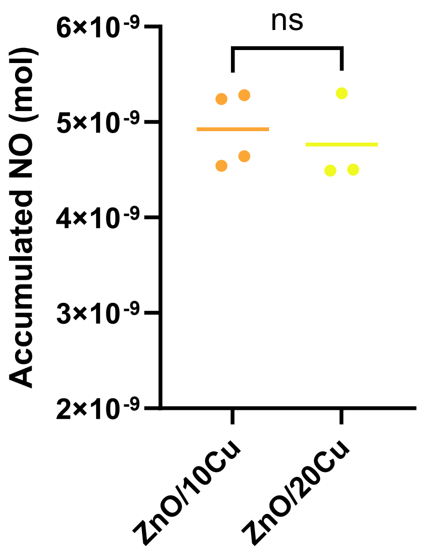

Figure S5. Nitric Oxide Generation Extension to 20 wt% Copper Formulation. Accumulated NO does not significantly increase with a sample that contains 20 wt% Cu. Therefore, ZnO/10Cu is the sample type with the highest percentage of Cu that will be used in following studies. $n \geq 3$; data presented as mean ± SD.

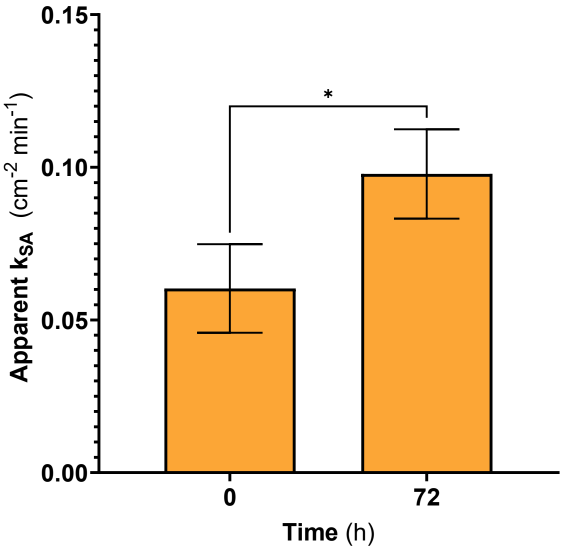

Figure S6. Stability of catalytic NO-generation following prolonged aqueous exposure. ZnO/10Cu coatings were immersed in PBS at 37 °C for 72 h prior to catalytic evaluation. The apparent surface area-normalized reaction rate constant (k_{SA}) was determined from the NO-generation profiles following exposure to 1 µM GSNO and 30 µM GSH. Fresh, non-incubated coatings (0 h) were included as controls. n≥3; data presented as mean ± SD. *$p < 0.05$

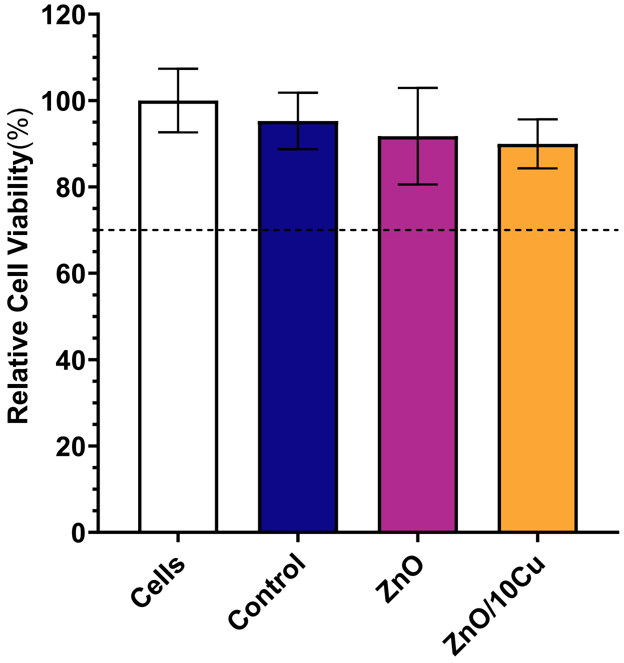

Figure S7. 24 h Leachate Cytocompatibility Assessment. Relative viability of 3T3 fibroblasts following 24 h exposure to leachates. All sample types maintained cell viabilities above the 70% ISO cytocompatibility threshold. n ≥ 4; data presented as mean ± SD.

Table S13. Statistical Significance (p-values) for t_{50} in Response to Material.

Formulation	Control	ZnO	ZnO/1Cu	ZnO/5Cu	ZnO/10Cu
**Control**		0.0532	0.0010	0.0003	<0.0001
**ZnO**			0.1038	0.0198	0.0009
**ZnO/1Cu**				0.8854	0.1798
**ZnO/5Cu**					0.6248
**ZnO/10Cu**					

Table S14. Cu Leaching Kit. Data normalized to various units and unit sizes for direct comparison.

Formulation	Cu (μM)	(μM cm^{-2})	(μg)	(μg cm^{-2})
		**24 h**		
**ZnO/1Cu**	2.72	2.09	0.173	0.133
**ZnO/5Cu**	3.91	3.00	0.248	0.191
**ZnO/10Cu**	8.71	6.70	0.554	0.426
		**72h**		
**ZnO/1Cu**	5.38	4.14	0.342	0.263
**ZnO/5Cu**	8.61	6.62	0.547	0.421
**ZnO/10Cu**	18.73	14.41	1.19	0.916

Table S15. Zn Leaching Kit. Data normalized to various units and unit sizes for direct comparison.

Formulation	Zn (μM)	(μM cm^{-2})	(μg)	(μg cm^{-2})
		**24 h**		
**ZnO**	1.66	1.27	0.105	0.081
**ZnO/1Cu**	1.21	0.93	0.077	0.059
**ZnO/5Cu**	1.25	0.96	0.079	0.061
**ZnO/10Cu**	1.27	0.98	0.081	0.062
		**72 h**		
**ZnO**	2.80	2.16	0.178	0.137
**ZnO/1Cu**	2.29	1.76	0.146	0.112
**ZnO/5Cu**	2.35	1.81	0.150	0.115
**ZnO/10Cu**	2.33	1.79	0.148	0.114

Table S16. Average *S. aureus* Log Difference in CFU cm^{-2}.

	Adhered					
**Formulation**	**Control (-)**	**Control (+)**	**ZnO (-)**	**ZnO (+)**	**ZnO/10Cu (-)**	**ZnO/10Cu (+)**
**Control (-)**		0.72	1.90	2.14	3.45	4.45
**Control (+)**			1.17	1.42	2.72	3.72
**ZnO (-)**				0.24	1.55	2.55
**ZnO (+)**					1.31	2.31
**ZnO/10Cu (-)**						1.00
**ZnO/10Cu (+)**						
	**Planktonic**					
**Formulation**	**Control (-)**	**Control (+)**	**ZnO (-)**	**ZnO (+)**	**ZnO/10Cu (-)**	**ZnO/10Cu (+)**
**Control (-)**		0.05	0.25	0.48	0.17	1.20
**Control (+)**			0.20	0.43	0.12	1.15
**ZnO (-)**				0.23	0.07	0.96
**ZnO (+)**					0.31	0.72
**ZnO/10Cu (-)**						1.03
**ZnO/10Cu (+)**						

Table S17. Statistical Significance (p-values) for *S. aureus* log(CFU cm^{-2}).

	Adhered					
**Formulation**	**Control (-)**	**Control (+)**	**ZnO (-)**	**ZnO (+)**	**ZnO/10Cu (-)**	**ZnO/10Cu (+)**
**Control (-)**		0.1196	<0.0001	<0.0001	<0.0001	<0.0001
**Control (+)**			0.0039	0.0006	<0.0001	<0.0001
**ZnO (-)**				0.9372	0.0002	<0.0001
**ZnO (+)**					0.0014	<0.0001
**ZnO/10Cu (-)**						0.0149
**ZnO/10Cu (+)**						
	**Planktonic**					
**Formulation**	**Control (-)**	**Control (+)**	**ZnO (-)**	**ZnO (+)**	**ZnO/10Cu (-)**	**ZnO/10Cu (+)**
**Control (-)**		>0.9999	0.8646	0.2859	0.9656	0.0004
**Control (+)**			0.9421	0.3953	0.9923	0.0006
**ZnO (-)**				0.8860	0.9993	0.0039
**ZnO (+)**					0.7224	0.0368
**ZnO/10Cu (-)**						0.0019
**ZnO/10Cu (+)**						

Table S18. Average *E. coli* Log Difference in CFU cm^{-2}.

	Adhered					
**Formulation**	**Control (-)**	**Control (+)**	**ZnO (-)**	**ZnO (+)**	**ZnO/10Cu (-)**	**ZnO/10Cu (+)**
**Control (-)**		0.13	0.72	0.83	2.31	2.68
**Control (+)**			0.59	0.70	2.18	2.55
**ZnO (-)**				0.11	1.59	1.96
**ZnO (+)**					1.48	1.85
**ZnO/10Cu (-)**						0.37
**ZnO/10Cu (+)**						
	**Planktonic**					
**Formulation**	**Control (-)**	**Control (+)**	**ZnO (-)**	**ZnO (+)**	**ZnO/10Cu (-)**	**ZnO/10Cu (+)**
**Control (-)**		0.03	0.04	0.04	0.04	0.52
**Control (+)**			0.01	0.01	0.01	0.49
**ZnO (-)**				0.00	0.00	0.48
**ZnO (+)**					0.00	0.48
**ZnO/10Cu (-)**						0.48
**ZnO/10Cu (+)**						

Table S19. Statistical Significance (p-values) for *E. coli* log(CFU cm^{-2}).

	Adhered					
**Formulation**	**Control (-)**	**Control (+)**	**ZnO (-)**	**ZnO (+)**	**ZnO/10Cu (-)**	**ZnO/10Cu (+)**
**Control (-)**		0.0920	<0.0001	<0.0001	<0.0001	<0.0001
**Control (+)**			<0.0001	<0.0001	<0.0001	<0.0001
**ZnO (-)**				0.2019	<0.0001	<0.0001
**ZnO (+)**					<0.0001	<0.0001
**ZnO/10Cu (-)**						<0.0001
**ZnO/10Cu (+)**						
	**Planktonic**					
**Formulation**	**Control (-)**	**Control (+)**	**ZnO (-)**	**ZnO (+)**	**ZnO/10Cu (-)**	**ZnO/10Cu (+)**
**Control (-)**		0.9706	0.9061	0.9557	0.9470	<0.0001
**Control (+)**			0.9998	>0.9999	>0.9999	<0.0001
**ZnO (-)**				>0.9999	>0.9999	<0.0001
**ZnO (+)**					>0.9999	<0.0001
**ZnO/10Cu (-)**						<0.0001
**ZnO/10Cu (+)**						

REFERENCES

(1) Koch, K.; Bhushan, B.; Jung, Y. C.; Barthlott, W. Fabrication of artificial Lotus leaves and significance of hierarchical structure for superhydrophobicity and low adhesion. *Soft Matter* **2009**, *5* (7), 1386-1393.
